# Serotonin 2C Receptor Agonists Improve Type 2 Diabetes via Melanocortin-4 Receptor Signaling Pathways

**DOI:** 10.1016/j.cmet.2007.10.008

**Published:** 2007-11-07

**Authors:** Ligang Zhou, Gregory M. Sutton, Justin J. Rochford, Robert K. Semple, Daniel D. Lam, Laura J. Oksanen, Zoe D. Thornton-Jones, Peter G. Clifton, Chen-Yu Yueh, Mark L. Evans, Rory J. McCrimmon, Joel K. Elmquist, Andrew A. Butler, Lora K. Heisler

**Affiliations:** 1 Department of Clinical Biochemistry, Addenbrooke's Hospital, University of Cambridge, Cambridge CB2 2QQ, UK; 2 Department of Internal Medicine, Yale University School of Medicine, New Haven, CT 06520, USA; 3 Pennington Biomedical Research Center, Louisiana State University System, Baton Rouge, LA 70808, USA; 4 Division of Endocrinology, Diabetes and Metabolism, Department of Medicine, Beth Israel Deaconess Medical Center, Boston, MA 02215, USA; 5 Department of Psychology, Sussex University, Brighton BN1 9QG, UK; 6 Department of Medicine, Addenbrooke's Hospital, University of Cambridge, Cambridge CB2 2QQ, UK; 7 Division of Hypothalamic Research and the Departments of Internal Medicine and Pharmacology, The University of Texas Southwestern Medical Center, Dallas, TX 75390-9051, USA

**Keywords:** HUMDISEASE, MOLNEURO

## Abstract

The burden of type 2 diabetes and its associated premature morbidity and mortality is rapidly growing, and the need for novel efficacious treatments is pressing. We report here that serotonin 2C receptor (5-HT_2C_R) agonists, typically investigated for their anorectic properties, significantly improve glucose tolerance and reduce plasma insulin in murine models of obesity and type 2 diabetes. Importantly, 5-HT_2C_R agonist-induced improvements in glucose homeostasis occurred at concentrations of agonist that had no effect on ingestive behavior, energy expenditure, locomotor activity, body weight, or fat mass. We determined that this primary effect on glucose homeostasis requires downstream activation of melanocortin-4 receptors (MC4Rs), but not MC3Rs. These findings suggest that pharmacological targeting of 5-HT_2C_Rs may enhance glucose tolerance independently of alterations in body weight and that this may prove an effective and mechanistically novel strategy in the treatment of type 2 diabetes.

## Introduction

The prevalence of type 2 diabetes has surged in recent decades. A growing body of work suggests not only that central neural pathways may play an important role in dysregulation of glucose homeostasis but also that these pathways are potentially amenable to therapeutic manipulation. Pharmacological compounds augmenting the tone of the neurotransmitter serotonin (5-hydroxytryptamine, 5-HT) have been extensively investigated for the treatment of obesity (Dourish, 1995; Simansky, 1996), a major risk factor for type 2 diabetes. However, the possibility of a direct role for serotonin in the pathophysiology and treatment of type 2 diabetes has received little attention.

Serotonin's effects on physiology and behavior are mediated by multiple serotonin receptors (5-HTRs) clustered into seven distinct families that are widely expressed in the central and/or peripheral nervous systems (Barnes and Sharp, 1999). Murine knockout studies reveal that only deletion of the gene encoding the 5-HT_2C_ receptor (5-HT_2C_R, formerly denoted 5-HT_1C_R) produces insulin resistance and type 2 diabetes, with antecedent hyperphagia and obesity (Bonasera and Tecott, 2000; Nonogaki et al., 1998; Tecott et al., 1995). These genetic studies demonstrate that 5-HT_2C_Rs are critical for energy homeostasis but do not indicate whether serotonin generally or 5-HT_2C_Rs specifically have a primary effect on glucose homeostasis that is dissociable from effects on body weight. Here, we examined whether 5-HT_2C_R agonists are effective in improving glucose tolerance in murine models of obesity and insulin resistance and whether these effects can be achieved in the absence of reductions in food intake and body weight.

## Results and Discussion

### 5-HT_2C_R Agonists Improve Glucose Homeostasis

**Figure 1:**
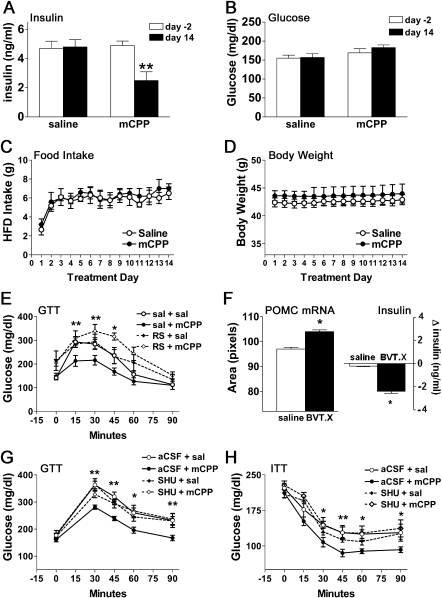
5-HT_2C_R Agonists Improve Glucose Homeostasis and Upregulate POMC mRNA without Affecting Food Intake or Body Weight (A–D) Treatment-induced changes in fasting plasma insulin and blood glucose, daily food intake, and body weight were assessed in diet-induced obese (DIO) mice (n = 10) treated with saline or mCPP (1 mg/kg/day). Compared to pretreatment levels (white bars, day −2), prolonged mCPP infusion (black bars, day 14) significantly reduced fasting plasma insulin (A) without altering blood glucose (B). This concentration of mCPP (•) had no discernable effect on food intake (C) or body weight (D) compared to saline treatment (○). (E) DIO mice (n = 12, mean body weight = 37 g) fasted for 12 hrs and then treated with a single injection of mCPP (1 mg/kg, i.p.; •) 45 min prior to a glucose load (1 g/kg, i.p.) showed significantly improved glucose tolerance compared to saline (○), an effect blocked by pretreatment with the 5-HT_2C_R antagonist RS102221 (RS; 1 mg/kg, i.p.) 30 min prior to mCPP treatment (◇). RS102221 alone (♦) had no significant effect on glucose tolerance. (F) *ob/ob* mice (n = 6, mean body weight = 52 g, mean blood glucose = 304 mg/dl) treated with BVT.X (40 mg/kg/day; black bar) for 7 days showed a significant enhancement of ARC POMC mRNA expression and a reduction in fasting plasma insulin levels compared to saline-treated counterparts (white bar). (G and H) A single injection of mCPP (1 mg/kg, i.p.; •) in 12 hr-fasted DIO mice (n = 36, mean body weight = 38 g) 45 min prior to a glucose (1 g/kg, i.p.) (G) or insulin (0.5 U/kg insulin) (H) load improved glucose and insulin tolerance compared to saline treatment (○), an effect blocked by pretreatment with the MC3R/MC4R antagonist SHU9119 (0.1 nmol, i.c.v.) 30 min prior to mCPP treatment (◇). SHU9119 alone (♦) had no significant effect on glucose or insulin tolerance. Data are presented as mean ± SEM. ^∗^p < 0.05, ^∗∗^p < 0.01.

To investigate whether 5-HT_2C_R stimulation affects glucose homeostasis, hyperinsulinemic diet-induced obese (DIO) male mice were treated with saline or a subanorectic dose of the classic 5-HT_2C_R agonist *m*-chlorophenylpiperazine (mCPP; 1 mg/kg/day) via subcutaneous (s.c.) osmotic minipump for 14 days. Baseline fasting plasma insulin and blood glucose were determined 2 days preceding pump implantation (day −2) and after 14 days of saline or mCPP infusion. While saline treatment did not significantly affect any parameter assessed, mCPP treatment produced a 49% reduction in fasting plasma insulin (Figure 1A; t(4) = 4.3, p < 0.01) without altering blood glucose (Figure 1B), food intake (high-fat diet [HFD]; Figure 1C), or body weight (Figure 1D).

We further characterized this effect using the *Lep^ob^* (*ob/ob*) murine model of severe obesity and insulin resistance, a second 5-HT_2C_R agonist, and another diet. Mice were individually housed in Comprehensive Lab Animal Monitoring System (CLAMS) chambers recording food intake, locomotor activity, and energy expenditure. Baseline and treatment-induced changes in fasting plasma insulin, fasting blood glucose, and % body fat were also measured. Hyperinsulinemic male *ob/ob* mice receiving a subanorectic dose of the selective 5-HT_2C_R agonist BVT.X (40 mg/kg/day) for 14 days exhibited a 45% improvement in fasting plasma insulin (see Figure S1A in the Supplemental Data available with this article online; t(6) = 3.8, p < 0.01) with no concomitant change in blood glucose (Figure S1B), food intake (powdered chow; Figure S1C), body weight (Figure S1D), % body fat (Figure S1E), locomotor activity (Figure S1F), or energy expenditure (VO_2_; Figure S1G). These data indicate that 5-HT_2C_R agonists ameliorate hyperinsulinemia in mild and severe obesity independently of alterations in food intake and body weight.

To establish whether 5-HT_2C_R activation enhances glucose tolerance, hyperinsulinemic drug-naive DIO mice were given a single injection of mCPP 45 min prior to a glucose tolerance test (GTT; 1 g/kg glucose, administered intraperitoneally [i.p.]). mCPP produced significant improvements in glucose tolerance (Figure 1E; F(3,9) = 488.2, p < 0.001). Pretreatment with the selective 5-HT_2C_R antagonist RS102221 (RS; 1 mg/kg, i.p.; Figure 1E) abolished this effect, confirming that mCPP-induced improvements in glucose tolerance are 5-HT_2C_R mediated.

### 5-HT_2C_R Agonists Upregulate POMC mRNA

5-HT_2C_Rs are expressed in multiple regions of the central nervous system (Molineaux et al., 1989). Of particular interest are a population of 5-HT_2C_Rs coexpressed with neurons producing the endogenous melanocortin receptor agonist α-melanocyte-stimulating hormone (α-MSH) in the arcuate nucleus of the hypothalamus (ARC) (Heisler et al., 2002). Pharmacological activation of central melanocortin pathways enhances insulin sensitivity, and overexpression of the precursor to α-MSH, pro-opiomelanocortin (POMC), attenuates insulin resistance in *ob/ob* mice (Heijboer et al., 2005; Mizuno et al., 2003; Obici et al., 2001). This led us to hypothesize that 5-HT_2C_R agonist-induced improvements in insulin sensitivity involve stimulation of POMC synthesis in the ARC. To address this possibility, *ob/ob* mice were treated with saline or BVT.X (40 mg/kg/day) via s.c. osmotic minipump for 7 days prior to densitometric quantification of ARC POMC mRNA with in situ hybridization histochemistry (ISHH). BVT.X produced a significant increase in the density of POMC mRNA in the ARC (Figure 1F, left; t(4) = 2.8, p < 0.05) in association with a significant reduction in fasting plasma insulin (Figure 1F, right; t(4) = 3.5, p < 0.05). These data suggest that 5-HT_2C_R agonist-induced improvements in hyperinsulinemia may be achieved through increased α-MSH availability.

### The Effect of 5-HT_2C_R Agonists on Glycemia Requires Central Melanocortin Receptors

To confirm that the effect of 5-HT_2C_R agonists on glucose tolerance is mediated by central melanocortin pathways, we investigated whether pharmacological blockade of central melanocortin-3 receptors (MC3Rs) and melanocortin-4 receptors (MC4Rs) hinders the ability of mCPP to enhance glucose tolerance. Specifically, hyperinsulinemic DIO mice were pretreated with the MC3R/MC4R antagonist SHU9119 (0.1 nmol, administered intracerebroventricularly [i.c.v.]) and then treated with mCPP (1 mg/kg, i.p.) prior to receiving a glucose bolus (1 g/kg glucose, i.p.). Once again, a significant improvement in glucose tolerance was seen after treatment with mCPP (Figure 1G; F(3,25) = 5.1, p < 0.01), an effect that was completely abrogated by pretreatment with SHU9119. The melanocortin antagonist in isolation had no discernible effect on glycemic excursion.

To assess insulin sensitivity more directly, we next performed an insulin tolerance test (ITT; 0.5 U insulin/kg, i.p.) in DIO mice and found that mCPP significantly enhanced the hypoglycemic response to insulin (Figure 1H; F(3,30) = 3.5, p < 0.05). This effect was fully reversed by i.c.v. pretreatment with SHU9119 (0.1 nmol, i.c.v.), which alone had no significant effect on insulin tolerance. Importantly, mice undergoing GTTs or ITTs did not have access to food, and the concentration of mCPP used did not affect appetite when tested in obese mice (Figure S2A). These findings confirm that 5-HT_2C_R agonist-induced improvements in glucose and insulin tolerance are dependent on central melanocortin pathways and are dissociable from effects on food intake.

### The Effect of 5-HT_2C_R Agonists Is Mediated Specifically via MC4Rs

**Figure 2:**
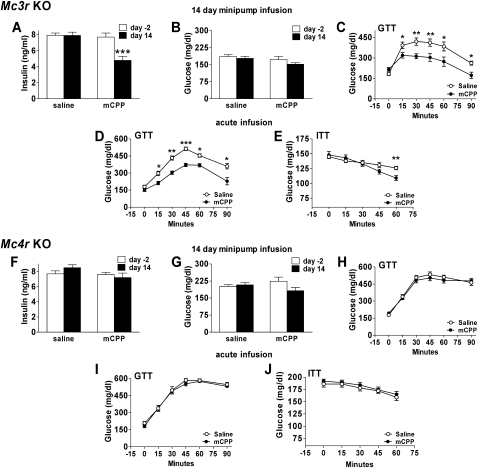
5-HT_2C_R Agonists Significantly Reduce Plasma Insulin and Improve Glucose Tolerance in *Mc3r* Knockout but Not *Mc4r* Knockout Mice (A–C) Compared to pretreatment levels (white bars, day −2), 14 days of mCPP (1 mg/kg/day; black bars, day 14) treatment in *Mc3r* KO mice (n = 10, mean body weight = 43 g) significantly reduced fasting plasma insulin (A) without altering fasting blood glucose (B) and significantly improved glucose tolerance (C) following a glucose load (1 g/kg, i.p). (D and E) A single injection of mCPP (1 mg/kg, i.p.; •) in treatment-naive 12 hr-fasted *Mc3r* KO mice (n = 12, mean body weight = 42 g) 45 min prior to a glucose (1 g/kg, i.p.) (D) or insulin (0.5 U/kg insulin) (E) load also improved glucose and insulin tolerance compared to saline treatment (○). (F–H) In contrast, *Mc4r* KO mice (n = 12, mean body weight = 60 g) treated with mCPP (black bars or •) showed no differences in plasma insulin (F), fasting blood glucose (G), or glucose tolerance (H) following a glucose load (1 g/kg, i.p.) compared to pretreatment values or saline treatment (white bars or ○). (I and J) A single injection of mCPP (•) was similarly ineffective in altering glucose tolerance following a glucose (1 g/kg, i.p.) (I) or insulin (0.5 U/kg insulin, i.p.) (J) load compared to saline (○) in treatment-naive *Mc4r* KO mice (n = 12, mean body weight = 59 g). Data are presented as mean ± SEM. ^∗^p < 0.05, ^∗∗^p < 0.01, ^∗∗∗^p < 0.001.

Efforts to discriminate the role of specific melanocortin receptors indicate that neural outputs important for maintaining insulin sensitivity are coupled to melanocortin tone primarily through MC4Rs (Fan et al., 2000; Heijboer et al., 2005; Huszar et al., 1997; Farooqi et al., 2000). We thus hypothesized that the mechanism underlying 5-HT_2C_R agonist-induced insulin sensitization involves increased activation of MC4Rs via upregulation of ARC POMC mRNA synthesis. We examined this hypothesis using obese and hyperinsulinemic *Mc4r* KO mice (Huszar et al., 1997) and *Mc3r* KO littermates (Butler et al., 2000) maintained on HFD to induce comparable obesity and hyperinsulinemia. Mice were treated via s.c. osmotic minipump with saline or mCPP (1 mg/kg/day) for 14 days. mCPP substantially reduced hyperinsulinemia (Figure 2A; t(4) = 4.3, p < 0.01) without affecting blood glucose (Figure 2B) and improved glucose tolerance (Figure 2C; F(1,7) = 387.2, p < 0.001) in *Mc3r* KO mice to the same extent observed in wild-type (WT) DIO mice (Figures 1A, 1B, 1E, and 1G), effects achieved without altering food intake (Figure S2B) or body weight (Figure S2C). Improvements in glucose tolerance (Figure 2D; F(1,7) = 387.2, p < 0.001) and insulin tolerance (Figure 2E; F(1,7) = 4.05, p < 0.05) were also observed in drug-naive *Mc3r* KO mice treated with a single injection of mCPP 45 min prior to a glucose (1 g/kg, i.p.) or insulin (0.5 U/kg, i.p.) load. In marked contrast, mCPP produced no discernable effect in *Mc4r* KO animals (Figures 2F–2J; Figures S2A, S2D, and S2E), supporting a specific role for the MC4R in mCPP-induced improvements in glucose tolerance.

### 5-HT_2C_R Agonists Activate Sympathetic Preganglionic Neurons via MC4Rs

**Figure 3:**
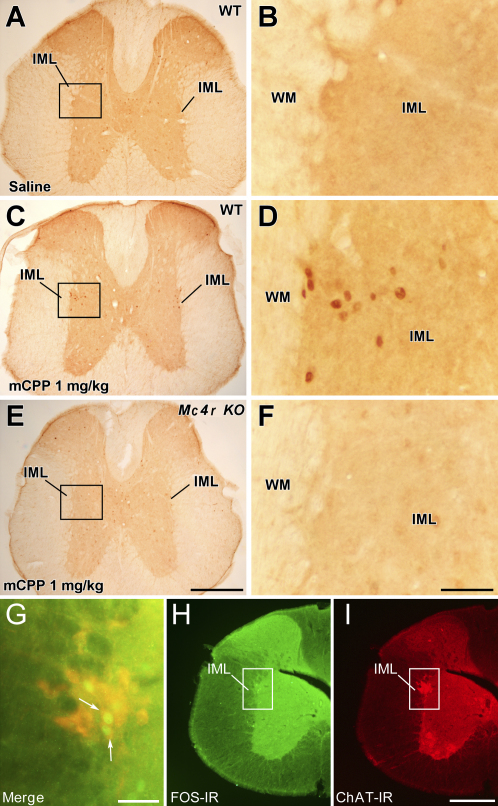
5-HT_2C_R Agonist-Induced Improvement in Glucose Tolerance Is Associated with MC4R-Dependent Increased FOS-IR in IML ChAT Neurons Brains from 12 hr dark-cycle-fasted wild-type (WT) and *Mc4r* KO littermates (n = 18; body weight range = 26–55 g) pretreated with saline or mCPP (1 mg/kg, i.p.) 45 min preceding a glucose bolus (1 g/kg, i.p.) were processed for immunohistochemistry. (A) FOS-IR was not evident in the intermediolateral nucleus of the spinal cord (IML) following saline treatment. (B) Magnification of boxed area in (A). (C–F) In contrast, FOS-IR was consistently evident in mCPP-pretreated WT mice (C and D), but not in *Mc4r* KO mice (E and F). (G–I) Subsequent analysis revealed that a substantial percentage of FOS-IR-positive neurons (H) (green fluorescent nuclear stain) were ChAT-IR positive (I) (red fluorescent cytoplasmic stain). (G) shows a merged and higher-magnification image of (H) and (I). White arrows indicate coexpression. Scale bar in (E) = 250 μM and applies to (A), (C), and (E). Scale bar in (F) = 25 μM and applies to (B), (D), and (F). Scale bar in (G) = 20 μM. Scale bar in (I) = 100 μM and applies to (H) and (I).

MC4Rs are expressed in brain regions containing both sympathetic and parasympathetic preganglionic neurons, each of which has been suggested to modulate glucose homeostasis (Kishi et al., 2003; Pocai et al., 2005). We investigated candidate populations of MC4Rs contributing to 5-HT_2C_R agonist-induced improvements in glucose tolerance using Fos-like immunoreactivity (FOS-IR) as a marker of neuronal activation in *Mc4r* KO mice and WT littermates treated with saline or mCPP (1 mg/kg, i.p.) prior to a glucose load (1 g/kg, i.p). Compared to saline treatment, mCPP induced a significant increase in FOS-IR in POMC neurons in the ARC of both *Mc4r* KO and WT mice (Figure S3; t(16) = 5.9, p < 0.001). POMC neurons have direct projections to the intermediolateral nucleus of the spinal cord (IML), which contains sympathetic preganglionic neurons expressing MC4Rs (Elias et al., 1998; Kishi et al., 2003). Consistent with enhanced sympathetic activity, we observed a significant increase in FOS-IR expression bilaterally in IML neurons of WT mice treated with mCPP compared to saline (Figures 3A–3D; Figure S4; t(16) = 4.2, p < 0.001), an effect absent in *Mc4r* KO mice (Figures 3E–3F; Figure S4).

Further examination indicated that mCPP induced FOS-IR in 91% of neurons in the IML staining for choline acetyltransferase (ChAT), the enzyme required for acetylcholine synthesis in sympathetic preganglionic neurons, in WT mice (Figures 3G–3I) but in <1% of ChAT neurons in *Mc4r* KO mice. In other regions associated with autonomic outflow, such as the paraventricular, ventromedial, dorsomedial, and lateral nuclei of the hypothalamus, the dorsal motor nucleus of the vagus (DMV), and the nucleus of the solitary tract, no difference in FOS-IR induction in response to mCPP treatment was observed between *Mc4r* KO and WT mice (Figure S5). We therefore conclude that IML MC4R activation is an important component of 5-HT_2C_R agonist-induced stimulation of the sympathetic nervous system (SNS). Nevertheless, potential caveats should be considered when interpreting these findings. For example, it is formally possible that MC4R deficiency affects basal and stimulus-induced activity specifically of IML sympathetic preganglionic neurons. It is also possible, given the widespread distribution of 5-HT_2C_Rs in the brain that include autonomic premotor sites, that administration of higher concentrations of 5-HT_2C_R agonists will activate non-MC4R-related pathways that provide inputs to preganglionic neurons influencing SNS activity. Thereby, conditions of MC4R-independent 5-HT_2C_R agonist-induced stimulation of the SNS would be achieved.

### 5-HT_2C_R Agonists Improve Peripheral Insulin Action via MC4Rs

**Figure 4:**
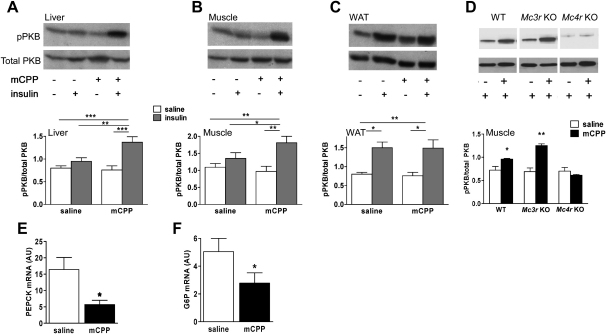
Effect of 5-HT_2C_R Agonists on Insulin Signaling (A–C) A single injection of mCPP (1 mg/kg, i.p.) 45 min prior to insulin (0.5 U/kg, i.p.; gray bars) significantly increased Ser473 phosphorylation of PKB in liver (A) and skeletal muscle (B), but not white adipose tissue (WAT) (C), compared to saline treatment in 12 hr-fasted DIO mice (n = 30, mean body weight = 37 g). Representative western blots are displayed above each bar graph. (D) Supporting a role for the MC4Rs in this effect, DIO WT and DIO *Mc3r* KO mice (n = 16, mean body weight = 42 g) treated with mCPP (1 mg/kg, i.p.; black bars) displayed a similar augmentation of insulin (0.5 U/kg, i.p.)-induced Ser473 phosphorylation of PKB in muscle compared to saline-treated counterparts (white bars), an effect absent in *Mc4r* KO mice. (E and F) Moreover, prolonged treatment with mCPP (1 mg/kg/day for 7 days, s.c. minipump) significantly reduced PEPCK (E) and G6P (F) mRNA expression in the liver of DIO mice (n = 10, mean body weight = 36 g). AU, arbitrary units. Data are presented as mean ± SEM. ^∗^p < 0.05, ^∗∗^p < 0.01, ^∗∗∗^p < 0.001.

To further characterize the effect of 5-HT_2C_R agonists on insulin action, we compared insulin-induced phosphorylation of protein kinase B (PKB) in quadriceps muscle, liver, and epididymal white adipose tissue (WAT) in DIO mice pretreated with saline or mCPP (1 mg/kg, i.p.) before administration of saline or insulin (0.5 U/kg, i.p). mCPP significantly enhanced PKB phosphorylation in response to insulin in liver (Figure 4A; F(3,26) = 9.0, p < 0.001) and muscle (Figure 4B; F(3,26), p < 0.01), but not WAT (Figure 4C), where significant insulin-induced PKB phosphorylation in the absence of mCPP (F(3,26) = 4.1, p < 0.05) may have precluded observation of a similar enhancement. To determine whether this effect is MC4R mediated, skeletal muscle was collected from insulinemia-matched DIO WT, DIO *Mc3r* KO, and *Mc4r* KO mice pretreated with saline or mCPP (1 mg/kg, i.p.) prior to an insulin load (0.5 U/kg, i.p.). mCPP significantly increased phosphorylation of PKB in both DIO WT (Figure 4D; t(4) = 3.8, p < 0.05) and DIO *Mc3r* KO (Figure 4D; t(5) = 5.6, p < 0.01) mice, an effect absent in *Mc4r* KO mice. These findings are concordant with the significant improvements in glucose tolerance, insulin tolerance, and fasting hyperinsulinemia seen in DIO WT and DIO *Mc3r* KOs, but not in *Mc4r* KOs, after mCPP treatment (Figure 1 and Figure 2). Furthermore, we also observed that prolonged treatment with mCPP (1 mg/kg/day for 7 days, s.c. osmotic minipump) significantly reduced levels of hepatic phosphoenolpyruvate carboxykinase (PEPCK; Figure 4E; t(8) = 3.8, p < 0.05) and glucose 6-phosphatase (G6P; Figure 4F; t(8) = 6.9, p < 0.05) mRNA compared to saline treatment, providing further evidence for hepatic insulin sensitization by 5-HT_2C_R agonist treatment.

Overall, these data support a model wherein 5-HT_2C_R agonists improve glucose homeostasis via increased production and release of endogenous melanocortin agonists, with subsequent activation of sympathetic preganglionic neurons in the IML via stimulation of MC4Rs. While we provide evidence for enhanced insulin sensitivity in response to 5-HT_2C_R agonists, it remains possible that the observed improved glucose tolerance is a composite of enhanced insulin secretion and enhanced insulin action in target tissues, though the relative contributions of these remain to be determined. The potential of 5-HT_2C_R agonists to address both cardinal pathogenic features of type 2 diabetes mellitus—insulin resistance and β cell failure—is intriguing.

In summary, the identification of new classes of antidiabetic agents is a clinical imperative. The findings presented here identify a novel therapeutic application for a class of pharmacological compounds developed more than two decades ago. We demonstrate that 5-HT_2C_R agonists significantly improve glucose tolerance and hyperinsulinemia in murine models of obesity and type 2 diabetes via an MC4R-dependent mechanism. These findings not only delineate specific neuronal pathways of relevance to a highly prevalent metabolic disease but also suggest that 5-HT_2C_R agonists may prove an effective and mechanistically novel treatment for type 2 diabetes.

## Experimental Procedures

### Subjects

Male *ob/ob* mice (B6.V-*Lep^ob^*/J), WT littermates, and DIO mice (Research Diets D12331i, 58% kcal fat) were purchased from The Jackson Laboratory. Male *Mc4r* KO (Huszar et al., 1997), *Mc3r* KO (Butler et al., 2000), and WT littermates were bred by mating heterozygotes. All mice were on a C57BL/6 background. Mice were individually housed with food (standard laboratory chow or HFD [Research Diets D12331i]) and water available ad libitum in a light- (12 hr on/12 hr off) and temperature- (21.5°C–22.5°C) controlled environment unless otherwise noted. Procedures used were in accordance with the guidelines for the care and use of animals established by the USA National Institutes of Health (NIH).

### Energy Balance and Basal Blood/Plasma Analysis

Saline, BVT.X (40 mg/kg/day), or mCPP (1 mg/kg/day) were infused using 7 or 14 day osmotic minipumps (Alzet, Durect Corporation) implanted between the scapulae of *ob/ob*, DIO WT, DIO *Mc3r* KO, or *Mc4r* KO mice. Ad libitum food intake and body weight were measured daily following the onset of the light cycle. Two days prior to pump implantation (day −2) and at the cessation of treatment (day 7 or 14), mice were fasted for 12 hr during the dark cycle, and blood glucose (Glucometer Elite, Bayer) and plasma insulin (Mercodia Rat Insulin ELISA, ALPCO) levels were determined according to manufacturer protocols.

### Glucose Tolerance Test and Insulin Tolerance Test

After a 12 hr dark-cycle fast, mice were treated with 1 g/kg glucose or 0.5 U/kg insulin. Blood was sampled from the tail vein, and GTTs and ITTs were performed as described previously (Sutton et al., 2006). Mice receiving 0.1 nmol SHU9119 or artificial cerebrospinal fluid (aCSF) prior to GTT or ITT were stereotaxically implanted 2 weeks earlier with an indwelling cannula aimed at the lateral ventricle (relative to bregma: AP = −0.34 mm, ML = 0.08 mm, DV = 0.16 mm below the dura) using methods described previously (Heisler et al., 2002).

### Western Blots

Following a 12 hr dark-cycle fast, mice were pretreated with saline or mCPP (1 mg/kg, i.p.); 45 min later, mice were treated with saline or insulin (0.5 U/kg, i.p.). Ten minutes after this, mice were sacrificed, and skeletal muscle (quadriceps), liver, and epididymal WAT were rapidly extracted and flash frozen. Tissue samples were then homogenized in lysis buffer, and following centrifugation, protein concentrations were determined in the supernatant using methods described previously (Sutton et al., 2006). Samples containing equal amounts of protein were denatured and analyzed by SDS-PAGE and western blotting using phospho-PKB and PKB antibodies (Cell Signaling Technology).

### RNA Isolation and Real-Time PCR Analyses

Total RNA was isolated from tissue samples using RNA STAT-60 (AMS Biotechnology) and quantified. Five hundred nanograms of each sample was reverse transcribed using random hexamers and M-MLV reverse transcriptase (Promega). Expression of G6P and PEPCK was determined by real-time PCR on an ABI 7900HT Sequence Detection System (Applied Biosystems) according to the manufacturer's instructions, and target values were normalized to 18S rRNA. Real-time PCR reagents were from Applied Biosystems.

### Neurohistochemical Analysis

#### Tissue Preparation and Histology

Mice under deep anesthesia were perfused transcardially with 0.9% saline followed by 10% neutral buffered formalin (Sigma). Brains were removed, immersed in 20% sucrose in phosphate-buffered saline (PBS; pH 7.0) for 18–36 hr at 4°C, sectioned coronally on a freezing-sliding microtome at 25 μm, and collected in five equal series.

#### Immunohistochemistry for FOS-IR

Mirroring the procedure used for the GTT, 12 hr dark-cycle fasted WT and *Mc4r* KO littermates were pretreated with saline or mCPP (1 mg/kg, i.p.) and treated with glucose (1 g/kg, i.p.) 45 min later. Brain tissue was collected 2 hr later. Standard immunohistochemistry methods (Elias et al., 1998; Heisler et al., 2002; Kishi et al., 2003) were used with a monoclonal antibody raised against rabbit c-Fos (1:10,000, Calbiochem), a biotinylated donkey anti-rabbit secondary antibody (1:500, Jackson ImmunoResearch Laboratories, Inc.), avidin-biotin complex (ABC; 1:250, Vector Laboratories), and a solution of 0.04% diaminobenzidine tetrahydrochloride (DAB; Sigma) and 0.003% hydrogen peroxide in PBS chromogen. Tissues were then mounted onto slides. Using cresyl violet-stained adjacent sections to clarify nuclear boundaries, FOS-IR signal throughout the rostrocaudal extent of the mouse brain and spinal cord was evaluated using a Zeiss Axioskop 2, and qualitative estimates of FOS-IR staining in specific brain regions were made. Quantitative comparisons of FOS-IR between saline- and mCPP-treated WT and *Mc4r* KO mice were made in the hypothalamus, DMV, and IML.

#### Dual Immunohistochemistry

Standard methods (Zhou et al., 2003) were used to visualize dual-fluorescence imaging of FOS-IR and α-MSH-IR or ChAT-IR. Briefly, adjacent sections of brain tissue were incubated with rabbit anti-c-Fos antibody (1:10,000, Calbiochem) and either sheep anti-α-MSH (1:5,000, Chemicon) or goat anti-ChAT (1:5,000, Chemicon) serum, a biotinylated donkey anti-sheep (α-MSH) or donkey anti-goat (ChAT) secondary antibody (1:500, Jackson ImmunoResearch Laboratories, Inc.), and a cocktail of Alexa Fluor 594-conjugated streptavidin (1:1,000, Molecular Probes) and Alexa Fluor 488-conjugated donkey anti-rabbit (1:300, Molecular Probes). Tissues were mounted onto Superfrost Plus (Fisher Scientific) slides. A green reaction product was obtained for Fos, and a red reaction product was obtained for α-MSH and ChAT. Single- and dual-labeled neurons were quantified using a Zeiss Axioskop 2 microscope.

#### ISHH and Densitometry Analysis

Visualization of POMC mRNA in ARC of *ob/ob* mice treated with saline or BVT.X (40 mg/kg/day for 7 days) was achieved with ISHH using methods described previously (Elias et al., 1998; Heisler et al., 2002; Kishi et al., 2003). Briefly, the POMC riboprobe was synthesized by in vitro transcription using a T3 polymerase (Ambion) in the presence of ^35^S-labeled UTP in accordance with the manufacturer's protocols. The ^35^S-labeled POMC cRNA probe was diluted in a hybridization solution and allowed to hybridize to tissue at 57°C. Following this, sections were incubated in a 0.002% RNase A solution and rinsed with decreasing concentrations of sodium citrate buffer in increasing temperatures (50°C–60°C). Tissues were then mounted onto slides and exposed to BioMax MR film (Kodak) for 48–72 hr. Slides were dipped in NTB-2 photographic emulsion (Kodak), exposed for 1 week at 4°C, and developed with Kodak fixer and D-19 developer according to the manufacturer's instructions. Slides were counterstained with thionin, dehydrated, and coverslipped. Comparisons of POMC mRNA following saline or BVT.X treatment were made by assessing the autoradiographic ARC ^35^S-labled POMC on film as measured with a light box, a digital camera interface, and NIH Image software. Determination of the level of bregma for each section of brain tissue represented on film was made by examining corresponding sections on the thionin-counterstained slides. ARC ^35^S-labeled POMC signal within each section was analyzed by computing the area of signal for each section throughout the rostrocaudal extent of the ARC minus background.

### Comprehensive Lab Animal Monitoring System

The concomitant effect of the 5-HT_2C_R agonist BVT.X (40 mg/kg/day for 14 days, s.c. minipump) on ad libitum food intake, locomotor activity, and oxygen consumption was assessed in male *ob/ob* mice housed in CLAMS chambers (Columbus Instruments). CLAMS chambers are 1 l rectangular acrylic cages with a source of powdered laboratory chow linked to a balance for food intake determination, a photobeam array surround for locomotor activity determination, and an indirect open-circuit calorimeter for oxygen consumption/respiratory exchange ratio data collection. Data are collected through a computer interface. Mice were acclimated to the chambers prior to the start of the study. Once stable feeding patterns comparable to home-cage conditions were evident, experimental treatment was initiated.

### Dual-Energy X-Ray Absorptiometry

To measure the effect of saline and the 5-HT_2C_R agonist BVT.X (40 mg/kg/day for 14 days, s.c. minipump) on body composition, % body fat was assessed via dual-energy X-ray absorptiometry (Lunar PIXImus, MEC Lunar Corp.) immediately before minipump implantation (day 0) and after minipump extraction (day 14) in *ob/ob* mice assessed in CLAMS chambers.

### Drugs

Mice were treated with 0.9% pyrogen-free saline, the 5-HT_2C_R agonists BVT.X (kindly provided by Biovitrum) or mCPP (Sigma), or the 5-HT_2C_R antagonist RS102221 (Tocris Cookson). Drugs were dissolved in 0.9% saline and administered i.p. in 10 μl/g of body weight or s.c. via osmotic minipumps. The MC3R/MC4R antagonist SHU9119 (Sigma) was dissolved in aCSF and administered i.c.v. at a concentration of 0.1 nmol in a 5 μl volume.

### Data Analysis

Food intake, body weight, plasma insulin, and blood glucose data were assessed via dependent t test or repeated-measures analysis of variance (RM ANOVA). For GTTs and ITTs, differences between treatments were assessed via RM ANOVA. Treatment-induced changes in FOS-IR expression in brain and spinal cord were analyzed via independent samples t test. PKB phosphorylation in response to insulin treatment in liver, muscle, and WAT was analyzed via independent samples t test or one-way ANOVA. Differences between treatments in the expression of POMC, PEPCK, and G6P mRNAs were analyzed via independent samples t test. Post hoc tests were performed using Tukey's test. t test results are presented as t(degrees of freedom subjects) = value of t, p value. ANOVA results are presented as F(degrees of freedom between groups, degrees of freedom within group) = value of F, p value. For all analyses, significance was assigned at the p ≤ 0.05 level. Data are presented as mean ± SEM.

## Supplemental Data

Document S1. Five Figures
