# Supplementary material for: Serotonin 2C Receptor Agonists Improve Type 2 Diabetes via Melanocortin-4 Receptor Signaling Pathways
Source: Cell Metab. 2007 Nov 7;6(5):398–405. doi: 10.1016/j.cmet.2007.10.008 (PMC2075535; doi:10.1016/j.cmet.2007.10.008)
Supplement: Document S1. Five Figures [file mmc1.pdf]

Cell Metabolism, Volume *6*

Supplemental Data

Short Article

Serotonin 2C Receptor Agonists Improve Type 2 Diabetes via Melanocortin-4 Receptor Signaling Pathways

Ligang Zhou, Gregory M. Sutton, Justin J. Rochford, Robert K. Semple, Daniel D. Lam, Laura J. Oksanen, Zoe D. Thornton-Jones, Peter G. Clifton, Chen-Yu Yueh, Mark L. Evans, Rory J. McCrimmon, Joel K. Elmquist, Andrew A. Butler, and Lora K. Heisler

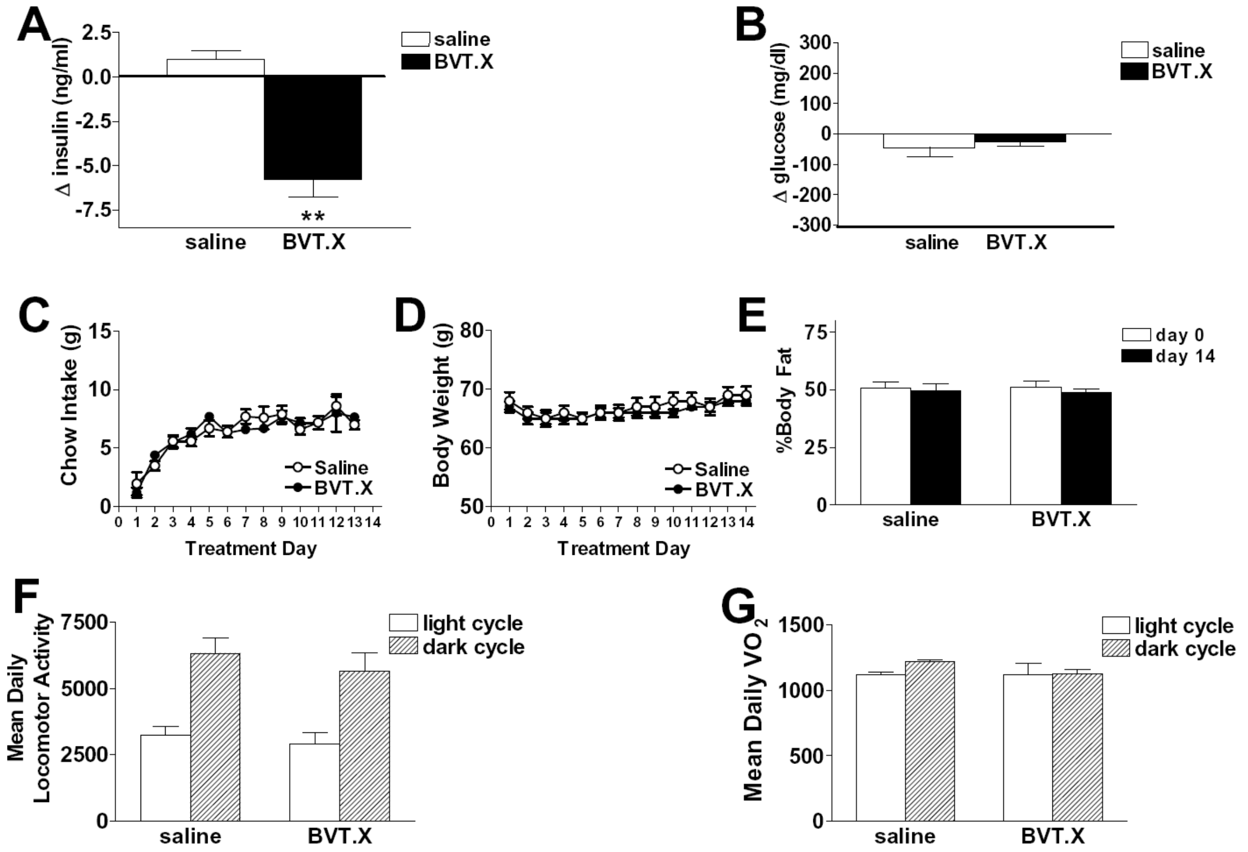

Figure S1. BVT.X Significantly Reduced Plasma Insulin without Affecting Blood Glucose, Food Intake, Body Weight, % Body Fat, Locomotor Activity, or Energy Expenditure

Male *ob/ob* mice (n = 7 per treatment, mean body weight = 68 g, mean fasting blood glucose = 268 mg/dl) individually housed in CLAMS chambers that produced continuous measures of food intake, activity, and energy expenditure were anesthetized, %body fat was determined using DEXA (day 0) and then mice were implanted with subcutaneous osmotic minipumps filled with saline or the 5-HT_{2C}R agonist BVT.X (40 mg/kg/day for 14 days).

(A and B) BVT.X treatment (black bars) significantly reduced fasting plasma insulin (12 hr dark cycle fast) by 45% from baseline (insulin day -2 = 12.7 ng/ml, day 14 = 6.9 ng/ml; $t(6)=3.8$, $p<0.01$) (A), but did not effect fasting blood glucose (B). Two weeks of saline treatment (white bars) did not significantly alter either fasting plasma insulin or blood glucose.

(C and D) Neither BVT.X (closed circles) nor saline (open circles) treatment affected chow intake (C) or body weight (D).

(E) No difference in %body fat were observed following 14 days of BVT.X or saline treatment (black bars) compared to pre-treatment levels (white bars).

(F and G) BVT.X also did not alter light (white bars) or dark (hatched bars) cycle locomotor activity (F), or light (white bars) or dark (hatched bars) cycle energy expenditure (as measured by mean daily VO_2) compared to saline treatment (G).

Data are presented as mean ± SEM, **$p<0.01$.

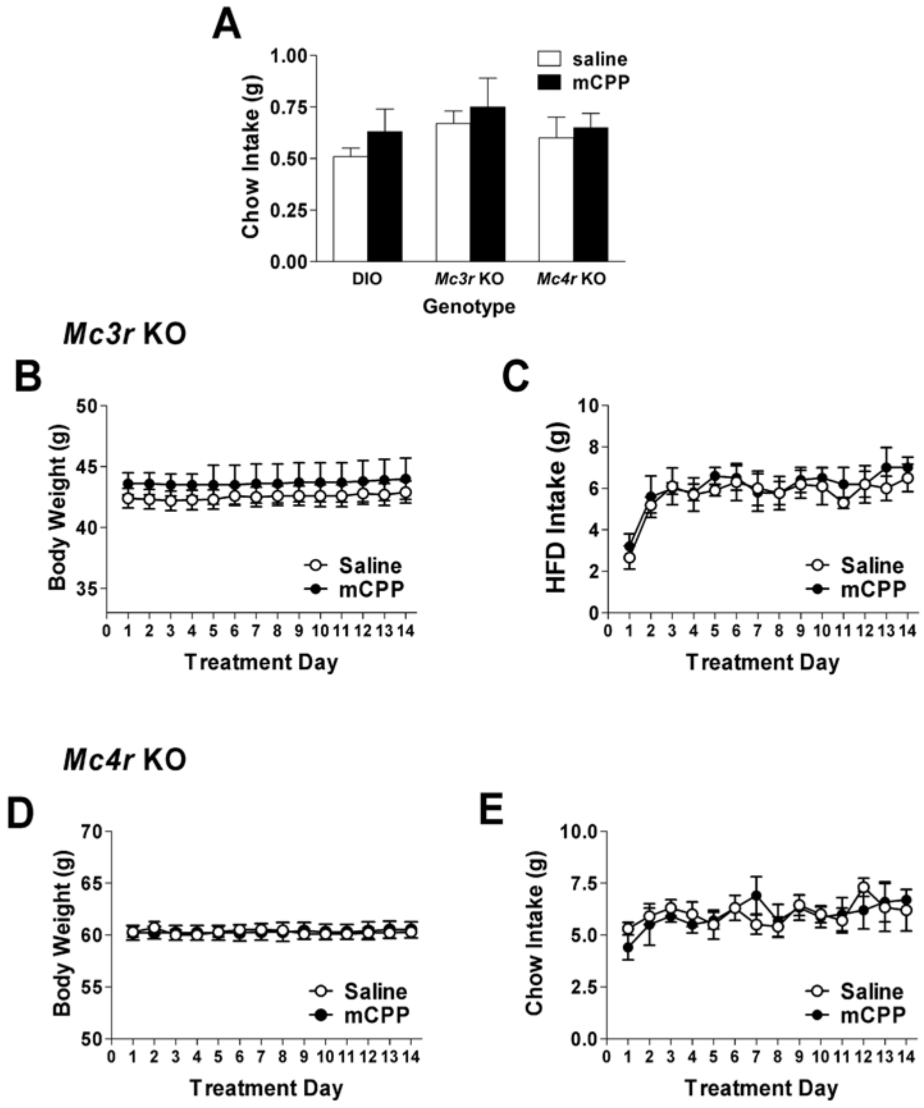

Figure S2. Confirmation that Acute and Chronic Administration of Subanorectic Dose of 5-HT$_{2C}$R Agonist mCPP Does Not Affect Food Intake or Body Weight in Mice

(A) Ad libitum fed male DIO (n = 12, mean body weight = 31 g), *Mc3r* KO (n = 12, mean body weight = 33 g), and *Mc4r* KO mice (n = 12, mean body weight = 37 g) mice exhibited no differences in dark cycle 2 hr chow intake when treated with either saline (white bars) or mCPP (1 mg/kg, i.p.; black bars).

(B-E) Like DIO mice (see Figure 1C-1D), *Mc3r* KO (B and C) and *Mc4r* KO mice (D and E) (n = 10-12 per genotype) showed no differences in daily food intake or body weight when implanted with a subcutaneous osmotic minipump filled with either saline (open circles) or a subanorectic dose of mCPP (1 mg/kg/day for 14 days, s.c.; closed circles).

Data are presented as mean ± SEM.

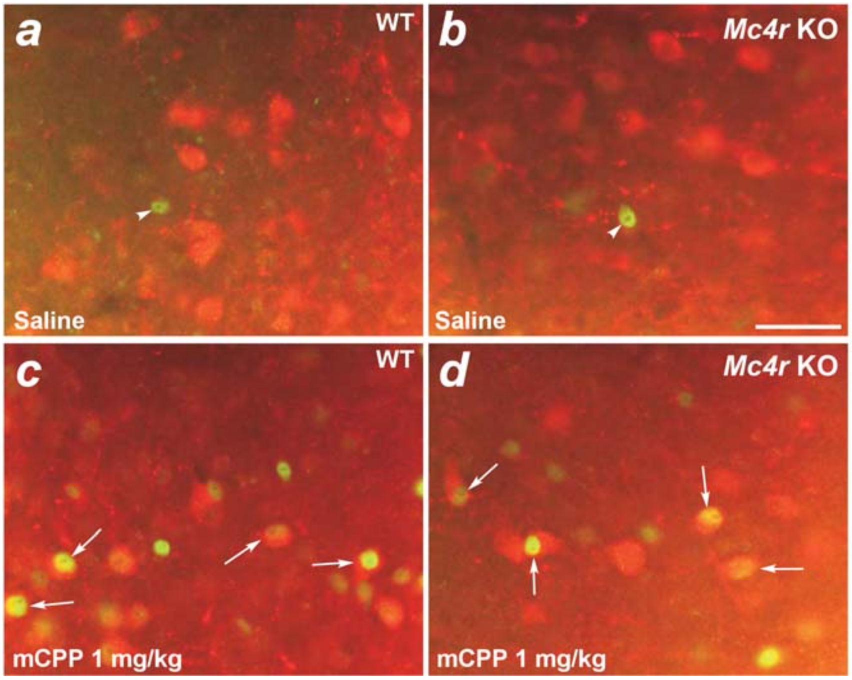

Figure S3. mCPP Pretreatment Prior to a Glucose Load Increased FOS-IR in ARC α-MSH Neurons in Both WT and *Mc4r* KO Mice

Adult male WT and *Mc4r* KO littermates (n = 9-10 per genotype; body weight range = 26-55 g) were deprived of food for 12 hrs during the dark cycle, then pretreated with saline or mCPP (1 mg/kg, i.p.), and then treated with glucose (1 g/kg, i.p.) 45 min later. Two hrs following this, mice were perfused with 0.9% saline and then 10% neutral buffered formalin under deep anesthesia, brains were extracted and processed for dual-label fluorescent histochemistry using rabbit anti-c-Fos and sheep anti-α-MSH primary antibodies. Compared to saline treatment (A and B), mCPP (C and D) increased the number of ARC FOS-IR (green fluorescent nuclear stain) in α-MSH positive (red fluorescent cytoplasmic stain) neurons in both WT and *Mc4r* KO mice (white arrows indicate co-expression). Specifically, mCPP induced FOS-IR in 53 ± 4% and 61 ± 3% of α-MSH neurons in WT and *Mc4r* KO mice, respectively. Scale bar in (B) represents 40 μM and applies to all panels.

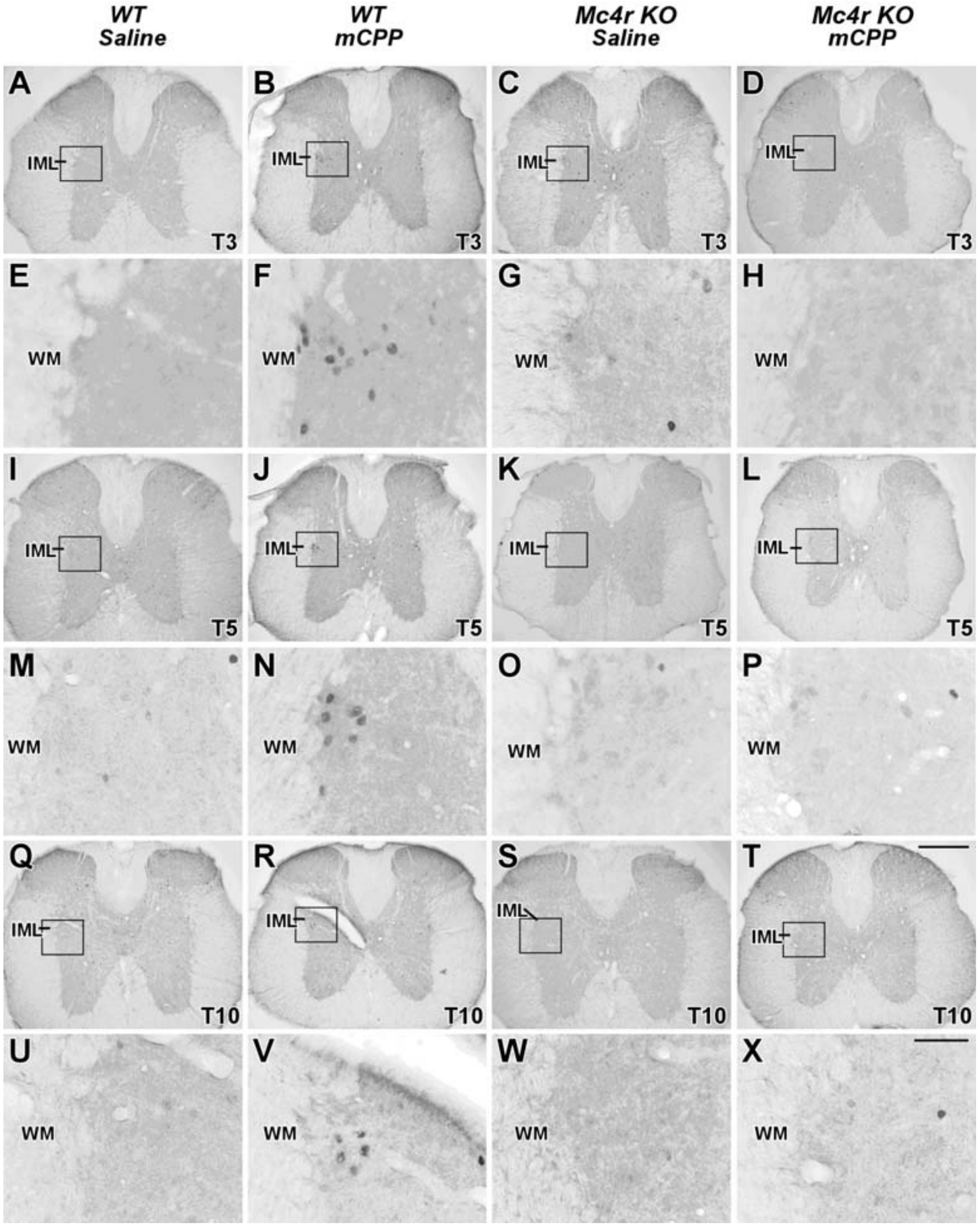

Figure S4. mCPP Pretreatment Prior to a Glucose Load Significantly Increased FOS-IR in the Intermediolateral Cell Column (IML) in WT, but Not *Mc4r* KO, Mice

Brain tissue from adult male WT and *Mc4r* KO littermates pretreated with saline or mCPP (1 mg/kg, i.p.) and treated with glucose (as described in Figure S3) was processed for FOS-IR (indicated by black nuclear stain) to investigate neuronal activation patterns in the IML, a region expressed at vertebral levels T1 - L2 which mediates sympathetic innervation of the body. Compared to saline treatment, mCPP increased FOS-IR in the IML of WT, but not *Mc4r* KO mice. Displayed are representative IML sections at level T3 (A-D, box enlarged in E-H), T5 (I-L, box enlarged in M-P), and T10 (Q-T, box enlarged in U-X). Scale bar in (T) represents 250 μM and applies to (A)-(D), (I)-(L), and (Q)-(T); scale bar in X represents 50 μM and applies to (E)-(H), (M)-(P), and (U)-(X).

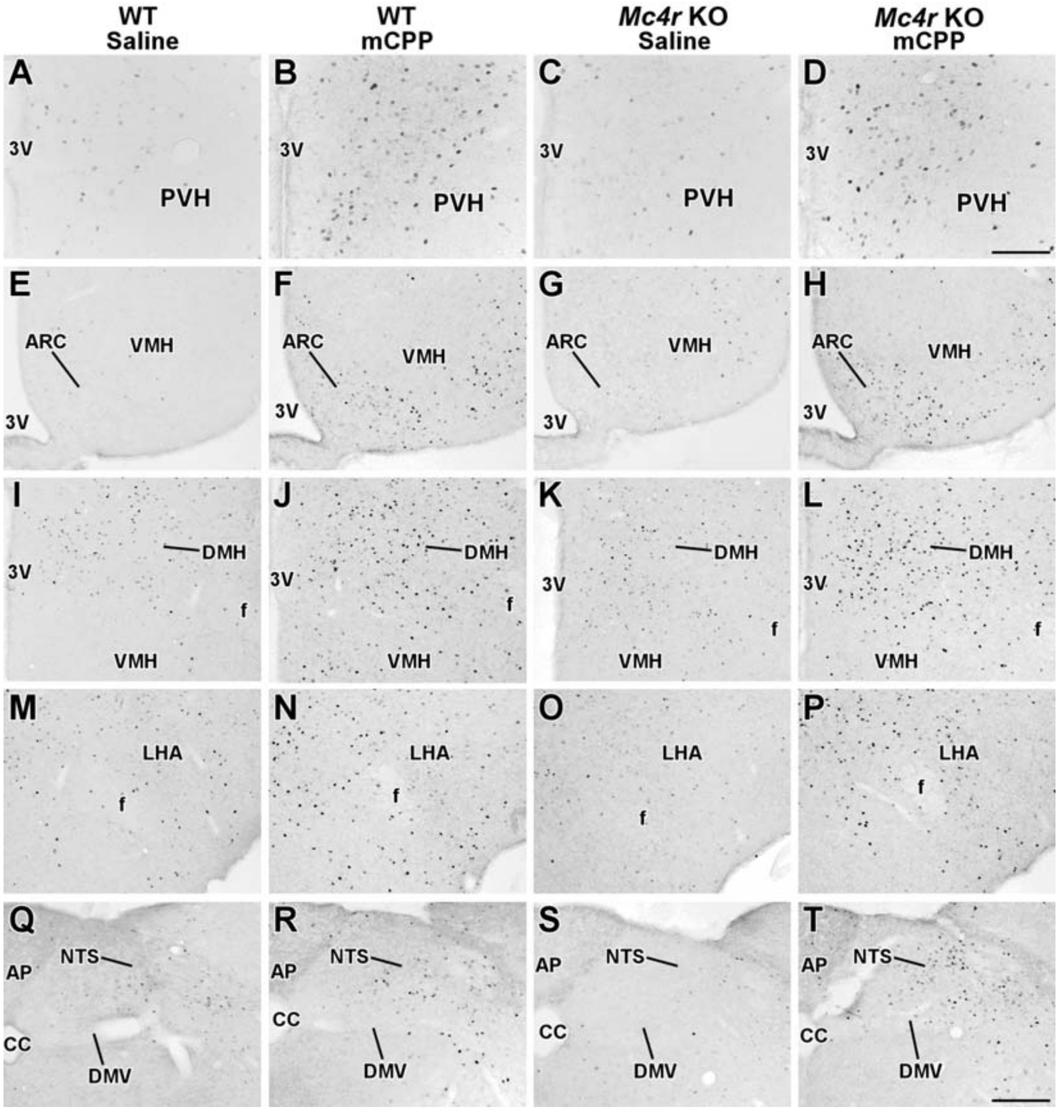

Figure S5. mCPP Pretreatment Prior to a Glucose Load Increased FOS-IR in Brain Regions Associated with Autonomic Outflow in Both WT and *Mc4r* KO Mice

Brain tissue from adult male WT and *Mc4r* KO littermates pretreated with saline or mCPP (1 mg/kg, i.p.) and treated with glucose (as described in Figure S3) was processed for FOS-IR (indicated by black nuclear stain) to investigate neuronal activation patterns in regions associated with autonomic outflow expressing MC4Rs. Compared to saline treatment, mCPP significantly increased FOS-IR in the paraventricular nucleus of the hypothalamus (PVH) (A-D), the arcuate nucleus of the hypothalamus (ARC) and ventromedial nucleus of the hypothalamus (VMH) (E-H), VMH and dorsomedial nucleus of the hypothalamus (DMH) (I-L), the lateral hypothalamic area (LHA) (M-P), and the dorsal motor nucleus of the vagus (DMV) and nucleus of the solitary tract (NTS) (Q-T) in both WT and *Mc4r* KO mice. These data are in contrast to those observed in the IML (Figure S4), and suggest that FOS-IR activation in these brain regions does not require functional MC4Rs. Scale bar in (D) = 50 µM and applies to (A)-(D)*; s*cale bar in (T) = 100 µM and applies to all other photomicrographs.
